# *Z*-scores outperform similar methods for analyzing CRISPR paralog synthetic lethality screens

**DOI:** 10.1186/s13059-025-03660-0

**Published:** 2025-07-02

**Authors:** Juihsuan Chou, Nazanin Esmaeili Anvar, Reem Elghaish, Junjie Chen, Traver Hart

**Affiliations:** 1 Department of Systems Biology, The University of Texas MD Anderson Cancer Center, Houston, TX USA; 2 The UT Health/MD Anderson Graduate School of Biological Sciences, Houston, TX USA; 3 Department of Experimental Radiation Oncology, The University of Texas MD Anderson Cancer Center, Houston, TX USA

## Abstract

Genetic screens offer a promising strategy for identifying tumor-specific therapeutic targets, but single-gene knockout screens often miss functionally redundant paralogs. Multiplex Cas9 and Cas12a CRISPR systems have been deployed to assay genetic interactions, but analysis pipelines vary considerably. Here we evaluate data from four in4mer CRISPR/Cas12a screens in cancer cell lines, using delta log fold change, *Z*-transformed dLFC, and rescaled dLFC approaches to identify synthetic lethal interactions. Both ZdLFC and RdLFC provide more consistent identification of synthetic lethal pairs across cell lines compared to the unscaled dLFC method, while ZdLFC benefits from not requiring a training set of known interactors.

**Supplementary Information:**

The online version contains supplementary material available at 10.1186/s13059-025-03660-0.

## Background

CRISPR-mediated genetic screens in over a thousand cell lines [1–4] have identified context-specific essential genes—candidate tumor-specific drug targets—but single-gene knockout screens systematically miss functional buffering by paralogs [5, 6]. To address this gap, several groups have developed Cas9- and Cas12a-based multiplex perturbation systems to assay genetic interactions in human cells. Each of five recent paralog synthetic lethality screens [6–10] uses conceptually similar approaches to identify genetic interactions, by comparing single-gene knockout phenotypes to paired knockout phenotype, but each group employs different hit-calling pipelines. Thompson et al. [8], Parrish et al. [7], Dede et al. [6], and Gonatopoulos-Pournatzis et al. [10] quantify genetic interaction effects by calculating the delta log fold change (dLFC), defined as the difference between observed and expected log2 fold change (LFC). The expected LFC for paired gRNA constructs is calculated by summing the observed LFC values for individual gRNAs paired with non-targeting controls [7, 8], intergenic controls [10], or nonessential controls [6], depending on the library design. From dLFC, hits are called using a variety of methods. Dede et al. [6] converted dLFC scores to *Z*-scores by fitting a normal distribution to a truncated distribution of observed dLFC scores, while other approaches include rank-sum tests [10], *t*-tests after variance smoothing [8], or regression-based methods [7]. Zamanighomi et al. employed GEMINI [11], a variational Bayesian method, to score GI, which we previously showed to offer no advantage over *Z*-scores in a limited screen [6].

While these methods for analyzing multiplex CRISPR screens have successfully identified robust interactions that withstand subsequent validation, their generalizability across different experimental designs has not been systematically tested. Here we compare several informatics pipelines for paralog synthetic lethality. We analyze data from paralog screens as described in Esmaeili Anvar et al. [12], a dataset independent of the previously described studies. In the absence of a widely accepted gold standard set of paralog synthetic lethals, we evaluate pipelines based on their ability to maximize consistency between experiments.

## Results and discussion

**Fig. 1:**
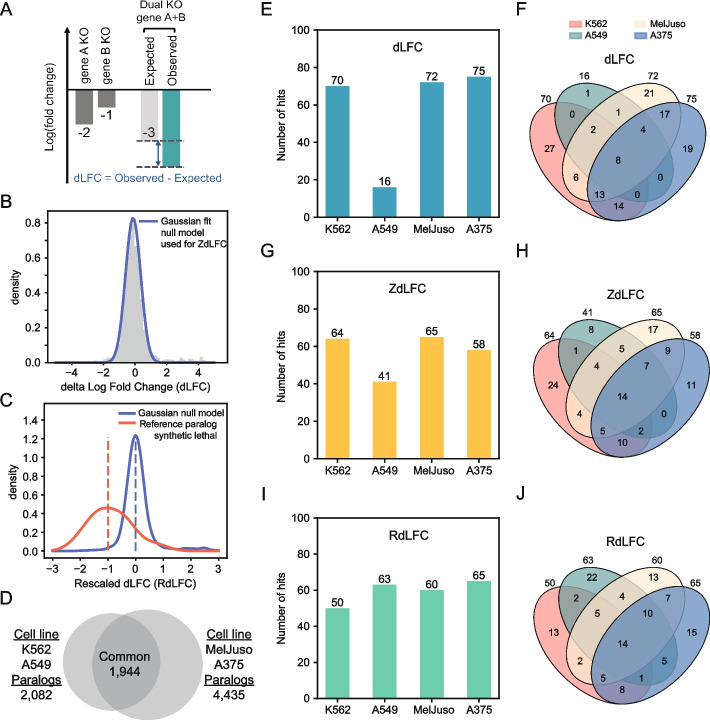
Measuring synthetic lethality between paralog pairs. **A** Single gene knockout (KO) fitness is determined by calculating the mean log fold change (LFC, y-axis) of gRNAs targeting the specific gene. The expected dual gene KO fitness is the sum of the single gene KO LFCs for gene A and gene B. The delta log fold change (dLFC) represents the difference between the observed and expected dual KO LFC. **B** The distribution of dLFC of all paralog pairs in an Inzolia screen is shown with a normal distribution fit (blue curve) after removing outliers (Methods). The normal fit estimates the null model, which is used to calculate the ZdLFC scores. **C** The dLFC distribution of an Inzolia screen after rescaling (Methods). The red and blue curves indicate kernel density estimates of the 13 reference paralog synthetic lethal (positive control) and the null model (negative control), respectively. The dotted lines indicate that the median of positive controls is rescaled to -1, while the negative controls are set to 0. **D** Four screens conducted with the Inzolia library: the “prototype” library in K562 and A549 cell lines, and the final Inzolia library in MelJuso and A375 cell lines, and the number of common paralog pairs between the two libraries. **E** Number of synthetic lethal hits in each screen identified using the dLFC method with a threshold of dLFC < -1 and LFC < -1. **F** Overlap of synthetic lethal hits identified in each of the four cell lines using the dLFC approach. **G**, **H** Number of synthetic lethals and their overlap with ZdLFC method (ZdLFC < -2, ZLFC < -2). **I**, **J** Number of synthetic lethals and their overlap with RdLFC < -0.7 and ZLFC < -2

Paralogs represent a systematic blind spot in monogenic CRISPR knockout screens as well as a fruitful search space in which to test genetic interaction technologies, both experimental and informatic, because functional buffering by paralogs is far more frequent than genetic interactions between non-paralogous genes. However, there is no clear standard for analyzing this type of data. Here we consider the most straightforward approach, delta log fold change (dLFC; Fig. 1A), and two derivatives of this approach, a *Z*-transformed ZdLFC and a supervised, rescaled RdLFC. dLFC represents the deviation of observed pairwise knockout effects from the expected additive effects of single knockouts. The ZdLFC refines this by fitting a Gaussian model to the dLFC distribution, estimating a null model, and scoring deviations from the null (high |*Z*| scores) as hits (Fig. 1B). The RdLFC uses the mean of this Gaussian fit as a null model (“0”) and adds an empirical model for hits (“ − 1”), and scales observed dLFC values according to these values, analogous to the CERES algorithm for gene essentiality in single-gene knockout screens [1] (Fig. 1C). Under this model, we use observed dLFC of the positive control reference set of 13 paralog synthetic lethals defined in Esmaeili Anvar et al. [12] (Methods). We also considered a fourth approach, ParaBagel, which derives a Bayes factor for synthetic lethality from these two models—analogous to the Bagel algorithm [13, 14] for classifying essential genes from CRISPR knockout screens—but we found this approach unsuitable for the data (Additional file 1: Supplementary methods). We apply these three approaches to data from two Inzolia library screens in cancer cell lines, and two additional screens with an earlier prototype version of the library (Fig. 1D; Esmaeili Anvar et al. [12]), to evaluate hit-calling consistency across pipelines. A complete set of scores for all pairs is available in Additional file 2: Table S1.

We applied these three methods to the 1944 paralog pairs that were common to the two in4mer libraries, across the four cell lines screened (Fig. 1D). As in Esmaeili Anvar et al. [12], we reasoned that most paralog synthetic lethality would be common across various backgrounds, and we therefore sought to identify the computational approach that maximized this commonality. We recognize that some synthetic lethal interactions—perhaps a large fraction—are context-specific, and we address several of these cases below. Moreover, even some “always synthetic lethal” paralogs may not be always synthetic lethal, if, for example, one gene in the pair is lost to mutation, epigenetic silencing, or genomic deletion in a specific tumor or tumor type. Nevertheless, in the absence of an accepted gold standard, we judge that maximizing common hits is a reasonable first-order approach for estimating accuracy of results.

Using the dLFC approach, with a simple threshold of dLFC <−1 (strong genetic interaction) and LFC of the pair <−1 (pairwise knockout shows strong fitness defect), we identified 16 to 75 paralog SL in the four cell lines (Fig. 1E), with considerable overlap (Fig. 1F). For ZdLFC, we defined a hit as ZdLFC <−2 and *Z*-transformed observed LFC <−2 (ZLFC, which uses a two-component Gaussian mixture model to fit the overall LFC distribution; Methods; Fig. 1G, H). Finally, for RdLFC, we defined a hit as interaction score <− 0.7 and same ZLFC <−2, with the interaction score threshold being somewhat arbitrarily chosen to yield roughly the same number of hits as the ZdLFC approach in order to minimize sample size as a source of bias when comparing the methods (Fig. 1I, J). A visualization of the three methods as applied to the four screens is available in Additional file 3: Fig. S1.

**Fig. 2:**
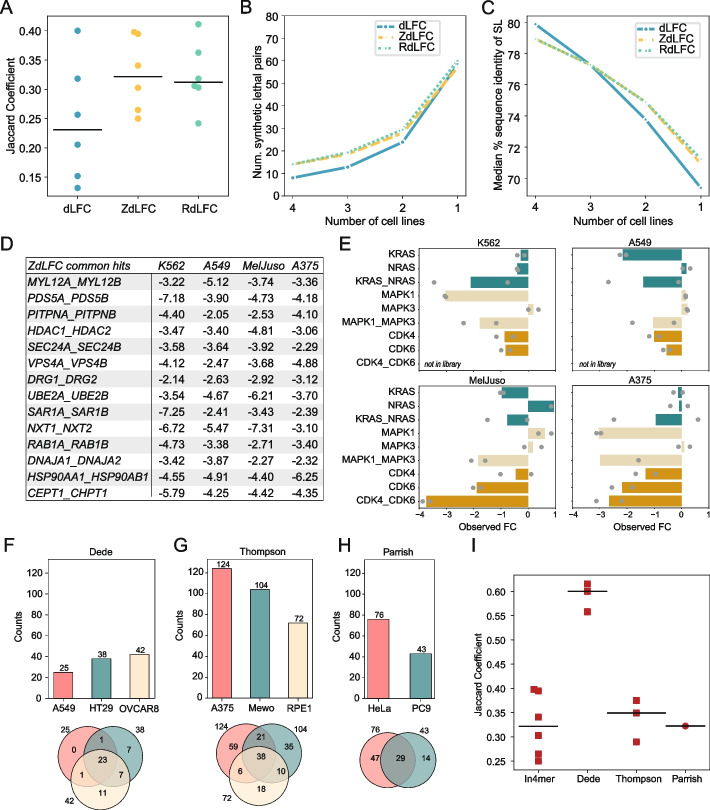
Relative performance (A) Jaccard coefficients comparing the hits across all pairs of Inzolia-screened cell lines using three different methods. Black line indicates the median Jaccard coefficient for each method. **B** Mean number of synthetic lethal pairs identified in all four, three, two, and one screens using the three methods. **C** Median percent sequence identity of all synthetic lethal pairs identified in all four, three, two, and one screens using the three methods. **D** ZdLFC score of common hits identified across all four screens. **E** Background-specific paralog synthetic lethals shown in all four cell lines. Gene pair *CDK4/CDK6* was not included in the prototype library. **F**, **G**, **H**) Number of synthetic lethal hits in each cell line and their overlap from three other studies: a Cas12a-based screen (Dede) and two SpCas9-based screens (Thompson, Parrish). **I** Jaccard coefficients comparing the hits across all pairs of cell lines within each study. Black line indicates the median of Jaccard coefficients for each study

To measure consistency of hits across cell lines, we calculated the Jaccard coefficient of all pairs of screens (Fig. 2A). With the thresholds we chose, the median Jaccard threshold for the ZdLFC approach is roughly equal to that of RdLFC, with both showing more consistency than the unscaled dLFC. We note that the ZdLFC < −2 threshold for pairwise knockout essentiality appears to maximize Jaccard similarity across the data sets, while the distribution of Jaccard coefficients is largely invariant to the secondary ZLFC threshold that specifies whether the dual knockout is itself essential (Additional file 3: Fig. S2A, B). Pairwise similarity of screens translates into groupwise similarity as well, as the number of hits in multiple screens is consistently higher with the *Z*-score and rescaling approaches (Fig. 2B) than the raw dLFC. The median pairwise sequence identity of synthetic lethals increases with the frequency of hits across cell lines (Fig. 2C), although the variation is low. However, the distribution of pairwise sequence identity of hits in two or three out of four screens is almost identical to that of hits in four out of four screens (Additional file 3: Fig. S2C). The Cohen’s *D* values are 0.06 and 0.03 for ZdLFC and RdLFC, respectively (hits in 3 vs. 4 cell lines), and 0.17 and 0.10 for 2 vs. 4 cell lines, with these very small effect sizes suggesting that sequence similarity might not be a good differentiator of context-dependent vs. pan-essential synthetic lethality. A list of top common synthetic lethals and their associated ZdLFC scores is shown in Fig. 2D; interestingly, only three gene pairs (*PITPNA/B*, *HDAC1/2*, *SAR1A/B*) intersect with the candidate positive control set we posited in Esmaeili Anvar et al. [12].

In contrast, hits with high pairwise sequence identity but observed in a single cell line contain clear examples of background-specific paralog synthetic lethals. For example, *NRAS/KRAS* are synthetic lethal in RTK-dependent cell line K562 while *KRAS* is singly essential in *KRAS* G12S mutant A549 cells (Fig. 2E), and *MAPK1/MAPK3* are synthetic lethal in A549 and MelJuso, while K562 and A375 show specific dependence on *MAPK1.* Finally, the *CDK4/CDK6* pair is strongly synthetic lethal in MelJuso, though the gene pair was not tested in K562 and A549. A full list of paralog scores for each method is available in Additional file 2: Table S1.

Given the above observations, we conclude that ZdLFC provides the best framework for scoring synthetic lethal paralogs. It provides more consistent hits across cell lines than the raw dLFC approach, in part because it can normalize for screens that show weaker overall distributions of fold change (e.g., A549 cells; Fig. 1E and Additional file 3: Fig. S1A). It yields virtually identical performance as the RdLFC (Fig. 2A–C), without requiring a training set of known positive interactors that must be included during screen library design.

We noted that the observed Jaccard coefficients for in4mer screens were significantly lower than those reported in Esmaeili Anvar et al. [12] for Cas12a-based paralog screens. We re-evaluated the Cas12a-based screen in Dede et al. [6] (Fig. 2F) and the two recent SpCas9-based paralog screens [7, 8] using the ZdLFC approach and found generally similar performance as previously reported (Fig. 2G, H), though Parrish et al. [7] (Fig. 2H) shows a marked improvement compared to our approach in Esmaeili Anvar et al. due to the screen-specific data normalization, as described in their study. It is worth noting that a Jaccard coefficient of 0.33 corresponds to an intersection encompassing 50% of each of two equal-sized sets and therefore indicates fairly strong coherence. Interestingly, the coherence of the Cas12a dual gRNA screens in Dede et al. [6] remains substantially above that of the other screens, while the Cas12a in4mer screens look very similar to the SpCas9 dual gRNA screens. The Dede et al. [6] test set was only 400 paralog pairs, while both Parrish et al. [7] and Thompson et al. [8] tested over 1000 gene pairs, suggesting the high coherence of the Dede et al. [6] data was strongly influenced by the selection of the paralogs to assay. Conversely, the in4mer screen re-analysis here encompasses nearly twice as many target pairs (*n* = 1944) and uses about fivefold fewer reagents per gene pair, and results in roughly the same level of coherence as the Cas9 screens.

Finally, we provide a summary of hits across the screening platforms considered here. Additional file 4: Table S2 contains an unweighted count of the number of screens in which each synthetic lethal is observed. We reasoned that the near-equal Jaccard coefficients of the in4mer [12], Parrish [7], and Thompson [8] platforms (Fig. 2I) and the apparent selection bias of Dede et al. [6] obviate the need for a weighted score as described in Esmaeili Anvar et al.

While prioritizing hit consistency across cell lines emphasizes recall, we also evaluated potential false positives and false negative rates. Both are difficult to evaluate given the lack of gold standards. For false positives, we examined the expression of synthetic lethal pairs identified in more than or equal to five screens (Additional file 4: Table S2), using RNA-seq data from the DepMap project [2, 15] (24Q4). We calculated the fraction of cell lines in which both genes in a pair were expressed (log_2_TPM > 2) across all 1673 24Q4 cell lines as well as within the 10 cell lines used in the in4mer [12], Dede [6], Parrish [7], and Thompson [8] studies (Additional file 3: Fig. S3A, B). Notably, 32 out of the 34 top hits had both genes expressed in more than 80% of cell lines, supporting the plausibility of these interactions and suggesting a background FDR of ~5%.

For false negatives, we are left with comparative data that, while indirect, collectively may point to a considerable false negative rate. First, there is relatively low overlap between the purported 13 gold standards of Esmaeili Anvar et al. and the subsequent in4mer screens, with only eight of the 13 called as hits in at least three of the four screens (Additional file 4: Table S2). Second, if a large fraction of paralog synthetic lethals are in fact common across most or all cell lines, we would expect a sensitive assay to yield a peak at hits in four cell lines relative to two or three (Fig. 2B). While these observations may point to a substantial number of false negatives, without more data, we cannot rule out the alternative hypothesis that these hits are indeed background-specific.

## Conclusions

Paralog synthetic lethality is of very high interest to the research community for several reasons. First, functional buffering by paralogs renders these gene families invisible to single-gene CRISPR knockout screens, revealing a knowledge gap in these large-scale efforts. Second, targeted therapies often inhibit related members of the same gene family, and in some cases rely on this multiple inhibition for efficacy (e.g., MEK, ERK, HDAC inhibitors), thus making multiplex genetic inhibition a requirement for modeling drug efficacy. Third, synthetic lethality and polypharmacology extend beyond paralogs, but the constrained search space and relative frequency of paralog synthetic lethals make this an ideal testing ground for developing informatic and experimental technology for more generalized approaches to genetic interaction.

To this end, we explored several bioinformatic options for analyzing paralog synthetic lethality data. We confirmed the importance of normalizing each data set, as has been the case for both monogenic and combinatorial CRISPR screens. Perhaps surprisingly, we find no benefit in using a training set of positive control synthetic lethals, although this may be simply because the training set is either too small or too noisy to be useful in this context. Nevertheless, it is clear that selecting thresholds based on implied negative controls, as in ZLFC/ZdLFC approach, gives more reproducible hits. Overall, we find that ~50% overlap between paralog synthetic lethal screens is a reasonable expectation for good quality screens at the current state of the art, depending on the composition of the set of genes being analyzed. Inevitably the 50% that does not overlap will contain some proportion of false positives that should be ignored, false negatives that should boost the overlap in a perfectly sensitive experiment, and true background-specific synthetic lethals. Until more primary and/or validation data is available, however, these will remain challenging to disambiguate.

Interestingly, the consistent ~50% overlap suggests that the Cas12a and the Cas9 genetic interaction platforms are roughly equivalent. Both implementations of the SpCas9 dual-promoter, dual-guide expression system gave similar results to the single-promoter, four-guide enCas12a system when applied to a large set of candidate paralogs. We did not evaluate the multi-Cas systems as they add another layer of complexity that, in our view, is not justified, given the capability of the other platforms.

## Methods

### Prototype and Inzolia screens using the in4mer platform

The prototype library consists of 43,972 arrays targeting 19,687 single genes, 2082 paralog pairs, 167 paralog triples, and 48 paralog quads. The screenings were conducted on two cancer cell lines, K562 and A549. The Inzolia library consists of 50,085 arrays targeting 19,687 single genes, 4435 paralog pairs, 376 paralog triples, and 100 paralog quads. Screenings were performed on the MelJuso and A375 cell lines. More details of paralog selection and library construction can be found in the in4mer paper [12].

The initial steps involved normalizing the raw read counts and assessing the overall quality of the screens. The read count data underwent preprocessing by adding a pseudocount of 5 reads to all arrays in each sample. The data was then normalized to a fixed total read count of 10 million reads. The guide-level log2 fold change (LFC) was calculated as the ratio of the normalized read count at the endpoint versus the T0 time point. Gene-level fold change (FC) was aggregated by averaging the guides. Both libraries included single knockout arrays targeting 50 essential genes as positive controls and 50 nonessential genes as the negative controls. These essential (CEGv2) and nonessential (NEG) genes were sourced from the Hart reference sets [16, 17]. The quality of the screens was evaluated using Cohen’s *D* score, calculated as the mean FC difference between the essential and nonessential controls divided by the pooled standard deviation (Additional file 3: Fig. S1A).

$$Quality\;Score=Cohen'sD=\frac{{meanLFC}_{nonessential}-{meanLFC}_{essential}}{pooled\;standard\;deviation}$$

### *Z*-transformed LFC

To calculate the *Z*-transformed LFC, the guide-level LFCs for each cell line were modeled using a two-component normal distribution with the GaussianMixture function from “sklearn.mixture” in Python. The distribution with the higher weight represented the majority of guides that did not affect fitness, while the distribution with the lower weight, smaller mean, and larger variance represented a smaller number of genes whose knockout increased fitness defects (Additional file 3: Fig. S1D). The mean and standard deviation of the higher-weight distribution were recorded and used to calculate the *Z*-transformed LFC [18]. The equation used is as follows:

$$Z-transformed\;{LFC}_{i,j}=\frac{{LFC}_{i,j}-\mu_{high\;weight,j}}{\sigma_{high\;weight,j}}$$

where *i* represents all arrays, including single genes and paralog pairs, *j* is the four cell lines, ${\mu }_{high weight, j}$ is the mean of the higher-weight distribution, and ${\sigma }_{high weight,j}$ is the standard deviation of the higher-weight distribution (Additional file 3: Fig. S1D).

### Three methods to score genetic interaction for paralogs

To identify the best method for detecting synthetic lethality in paralog screens, we analyzed the 1944 common paralog pairs from the prototype and Inzolia screens using three different quantification methods.

1. Delta log fold change (dLFC): Genetic interactions were quantified by calculating the delta log fold change (dLFC), which is the log fold change of the pairwise gene knockout (observed) minus the sum of the single-gene knockout log fold changes (expected) (Fig. 1A). This is referred to as the raw dLFC.
2. *Z*-transformed dLFC (ZdLFC): To enhance accuracy, we calculated the *Z*-transformed dLFC (ZdLFC). We sought the best null model to fit the dLFC distribution by testing various components and determined that a single Gaussian component provided the best fit. However, we noticed the presence of outliers. To address this, we experimented with removing different quantiles and applied the standard outlier removal method: Q1 − 1.5*IQR, Q3 + 1.5*IQR. This method yielded the smallest mean distance between the empirical distribution function and the cumulative distribution function (CDF) fitted to our real data after outlier removal. Therefore, we used the fitted normal distribution, excluding outliers as defined by Q1 − 1.5*IQR, Q3 + 1.5*IQR as our null model.
  $$Z-transformed\;{dLFC}_{k,j}=\frac{{dLFC}_{k,j}-\mu_{null\;model,j}}{\sigma_{null\;model,j}}$$
  where *k* represents all common pairs. Q1 is the 0.25 quantile of dLFC distribution, Q3 is the 0.75 quantile of dLFC distribution, and IQR = Q3 − Q1. ${\mu }_{null model, j}$ and ${\sigma }_{null model,j}$ are the mean and standard deviation of the null model for cell line *j* (Fig. 1B; Additional file 3: Fig. S1B).
3. Rescaled dLFC (RdLFC): The supervised RdLFC utilized the same null model as defined above. Additionally, it incorporated the observed dLFC of the 13 paralog synthetic lethal gold standards defined in Esmaeili Anvar et al. [12] as a positive control reference set. We rescaled the dLFC of all pairs by setting the median of the positive control set to − 1 and the mean of the null model to 0, adjusting the rest of the pairs accordingly.

$$Rescaled {dLFC}_{k, j}=\frac{{dLFC}_{k, j}-{\mu }_{null model, j}}{{\mu }_{null model, j}-median(\left\{{dLFC}_{k, j}: k \epsilon REF SL\right\})}$$

where *k* represents all common pairs, *j* represents the four cell lines, and *REF SL* represents the 13 paralog synthetic lethal gold standards (Fig. 1C; Additional file 3: Fig. S1C).

### Evaluation of the three methods

To identify the most consistent method for calling synthetic lethal hits, we calculated the Jaccard similarity coefficient. We first generated all combinations of the four cell lines. Next, for each pair of cell lines, we calculated the fraction of the intersection of hits over the union of hits. The final Jaccard coefficient for each method was the median of all Jaccard coefficients from these pairwise comparisons.

$$Jaccard\;coefficient\left(A,B\right)=\frac{\left|A\cap B\right|}{\left|A\cup B\right|}=\frac{\left|A\cap B\right|}{\left|A\right|+\left|B\right|-\vert A\cap B\vert}$$

We also calculated the percent sequence identity of pairs identified as hits. Percent sequence identity data were obtained from BioMart. For each gene pair, the percent identity of paralogs AB and BA was recorded separately. We calculated the mean percent sequence identity for each gene pair (Fig. 2C; Additional file 3: Fig. S2C).

### Re-analysis of prior work

To re-evaluate the Cas12a-based screen in Dede et al. [6] and the two SpCas9-based screens [7, 8] using the ZdLFC method, we followed these steps: first, raw read counts from the three studies were downloaded. The same preprocessing pipeline described above was applied to calculate gene-level LFC and dLFC. In Dede et al., the library targeted 403 paralog pairs and the screen was conducted in three cell lines: A549, HT29, and OVCAR8. The Thompson et al. study looked at 1191 paralog and non-paralog pairs in the A375, Mewo, and RPE cell lines. Parrish et al. targeted 1030 paralog pairs in HeLa and PC9 cell lines.

All LFC values in all screens were *Z*-transformed, and the dLFC values were *Z*-transformed as well. We used the same thresholds (ZdLFC < −2 and ZLFC < −2) to call hits in each study for fair comparison. To evaluate the consistency of hit calling across each method, we calculated the Jaccard coefficients (Fig. 2IG).

## Supplementary Information

Additional file 1: Supplementary methods.

Additional file 2: Table S1.

Additional file 3: Supplementary figures.

Additional file 4: Table S2.

## Data Availability

The datasets generated during the current study, along with all Python notebooks used for data analysis, are available in the Figshare repository at 10.6084/m9.figshare.28195865.v1 [19] .
