# Supplementary material for: Z-scores outperform similar methods for analyzing CRISPR paralog synthetic lethality screens
Source: Genome Biol. 2025 Jul 2;26:188. doi: 10.1186/s13059-025-03660-0 (PMC12217507; doi:10.1186/s13059-025-03660-0)
Supplement: Supplementary file 3 — Additional file 3: Supplementary figures. [file 13059_2025_3660_MOESM3_ESM.docx]

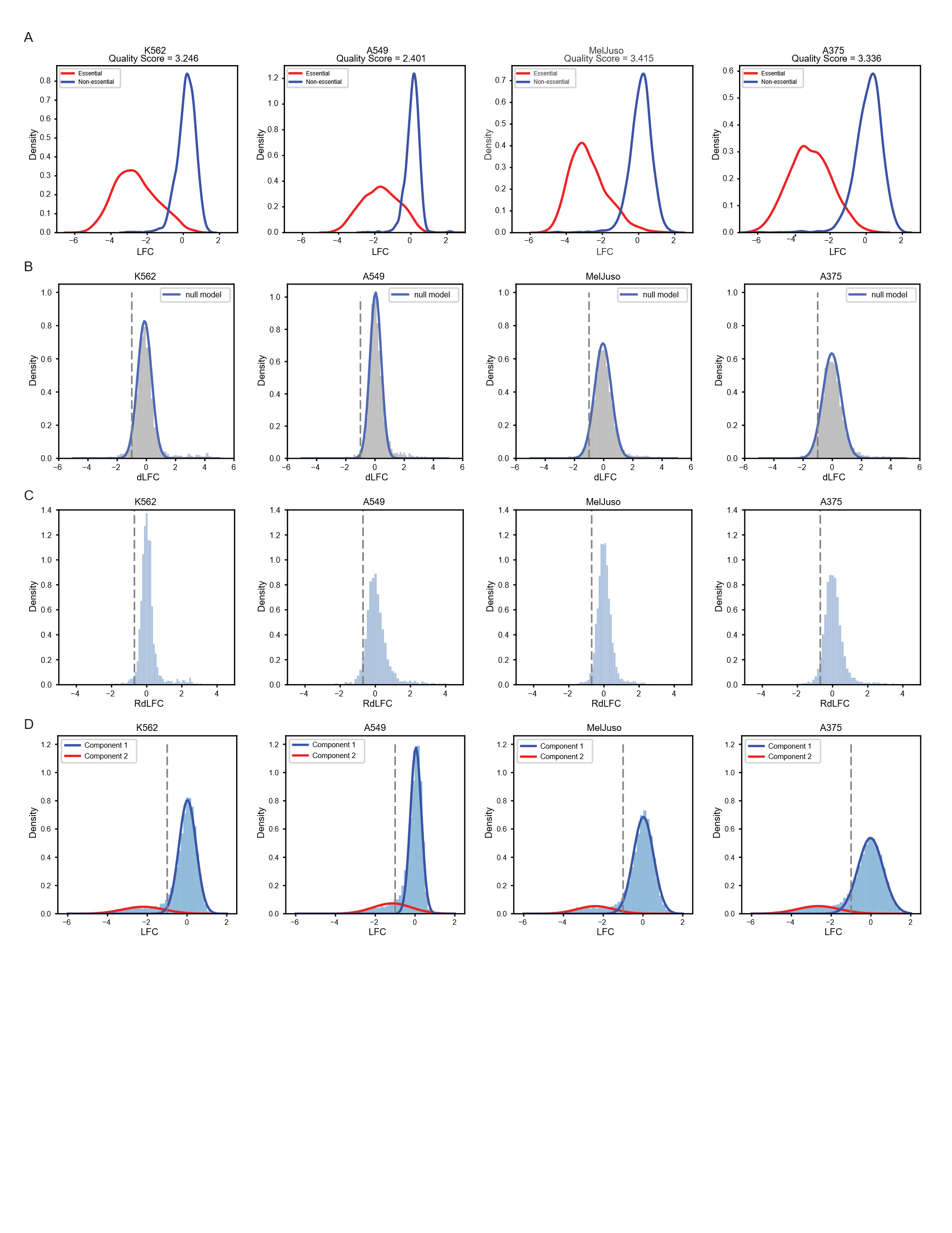


**Figure S1.** (A) LFC distributions of arrays targeting reference essential (red) and non-essential (blue) genes, along with Cohen’s D quality score in four cell lines. (B) dLFC histograms for the four cell lines with normal distribution fits after removing outliers (Methods). Gray dashed line, hit calling threshold for dLFC. Blue curve, Gaussian fit for calculating ZdLFC. (C) Distribution of RdLFC scores for each cell line, with grey dashed lines indicating hit calling threshold (RdLFC < 0.7). (D) Raw LFC histograms for the four cell lines, overlaid with weighted density curves from a two-component Gaussian Mixture model. The red component models the essential genes, while the blue component represents the majority of genes that do not show severe fitness defects and is used to calculate ZLFC.


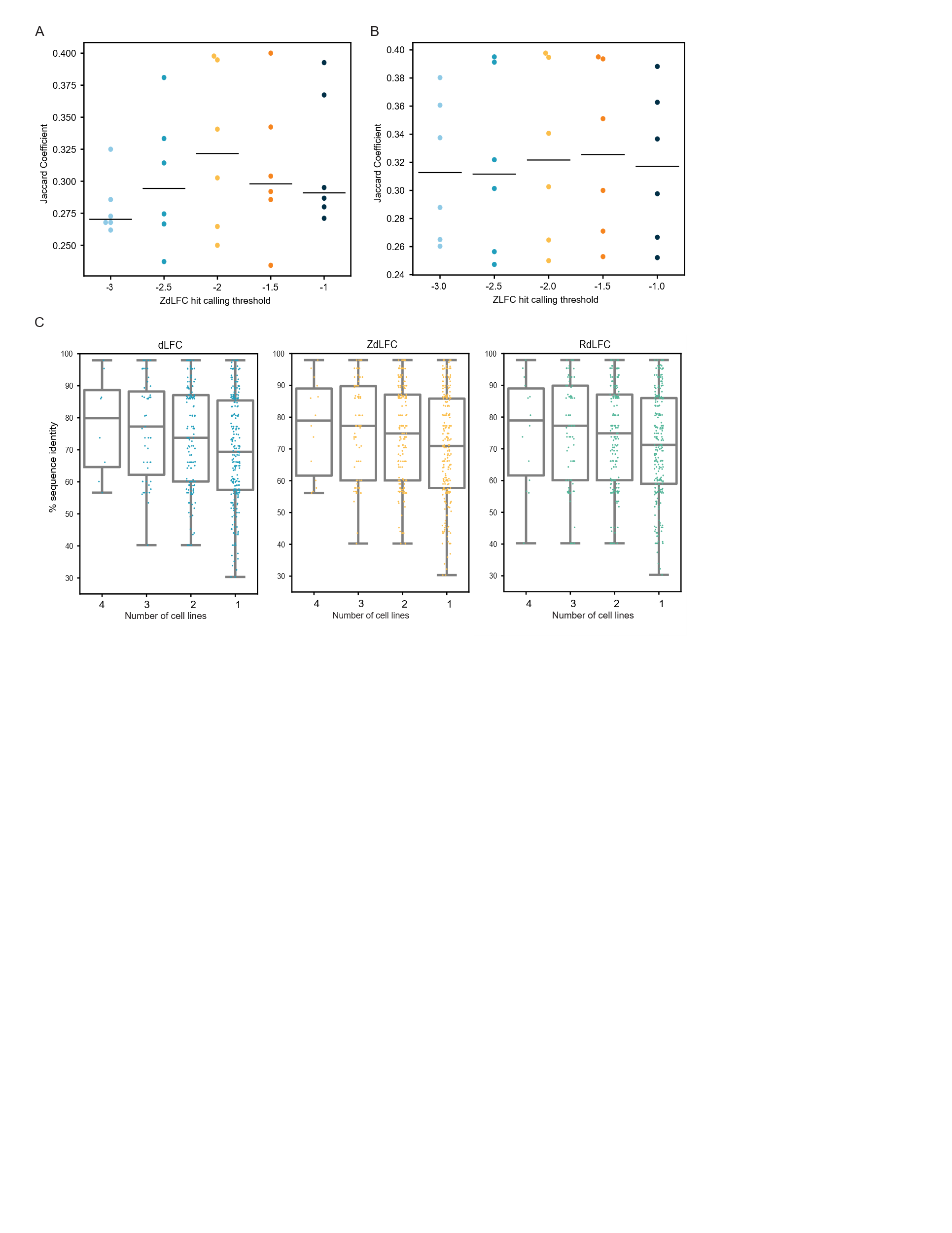


**Figure S2. Robustness of hit calling thresholds.** (A) Jaccard coefficients across all cell line pairs using different ZdLFC thresholds to call hits, with ZLFC threshold fixed at -2. Black line, median Jaccard coefficient. (B) Jaccard coefficients across all cell line pairs using different ZLFC cutoffs to call hits, with ZdLFC threshold fixed at -2. (C) Distribution of amino acid similarity among all synthetic lethal pairs identified in all four, three, two, and one screens using the three methods.


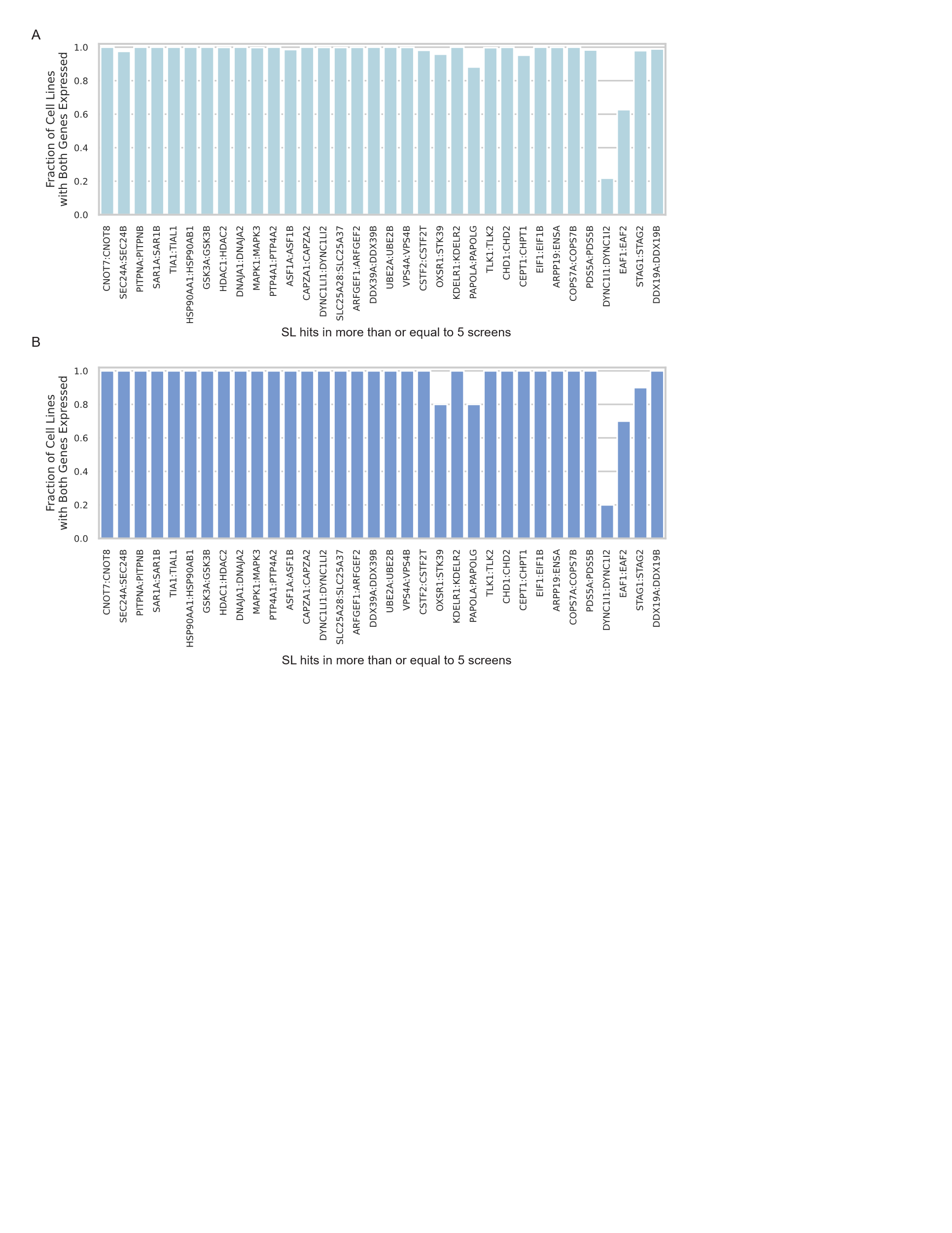


**Figure S3.** (A) Fraction of DepMap 24Q4 cell lines (n = 1,673) in which both genes of each synthetic lethal pair (identified in $\geq$ 5 screens; Supplementary Table 2) are expressed (log₂TPM > 2). (B) Same analysis as in (A), restricted to the 10 cell lines used in the in4mer^12^, Dede^6^, Parrish^7^, and Thompson^8^ screens.
